# Social determinants of health and periodontal disease in Brazilian adults: a cross- sectional study

**DOI:** 10.1186/1472-6831-13-22

**Published:** 2013-05-20

**Authors:** Maria de Lourdes Carvalho Bonfim, Flavio Freitas Mattos, Efigênia Ferreira e Ferreira, Ana Cristina Viana Campos, Andréa Maria Duarte Vargas

**Affiliations:** 1 Department of Dentistry, State University of Montes Claros, Montes Claros, MG, Brazil; 2 Department of Community and Preventive Dentistry, School of Dentistry, Federal University of Minas Gerais, Belo Horizonte, MG, Brazil; 3 Postgradute Program in Dentistry, Department of Community and Preventive Dentistry, School of Dentistry, Federal University of Minas Gerais, Belo Horizonte, MG, Brazil

**Keywords:** Social determinants of health, Periodontal disease, Oral health

## Abstract

**Background:**

Recently, increasing importance has been placed on the social determinants of health and disease. The present study aimed to determine the prevalence of periodontal disease in Brazilian adults and identify possible relationships with social determinants.

**Methods:**

A cross-sectional study was performed using a sample of 743 adults (aged 35–49 years) living in an urban area of a large city in southeastern Brazil. The condition of the periodontium was assessed using the Community Periodontal Index (CPI) according to the diagnostic criteria established by the World Health Organization (WHO). The variables related to social determinants were collected using a structured questionnaire. A descriptive analysis of all study variables was performed. Multiple correspondence analysis was subsequently performed to identify relationships between periodontal disease and the social determinants of health.

**Results:**

The periodontal exams showed that 36.5% of adults had a healthy periodontium, 2.0% had gingival bleeding, 47.1% had calculus and 9.5% had periodontal pockets of 4–5 mm. Periodontal pockets of 6 mm or more were the worst periodontal condition found (affecting only 2.1% of the participants). The correspondence analysis enabled us to form three groups with different profiles. The first group was distinguished by the presence of bleeding (gingivitis) or a healthy periodontium. The members of this group were typically aged 35 to 39 years and had 9–12 years or more than 12 years of education. The second group consisted of subjects with calculus and periodontal pockets of 4–5 mm. The members of this group were typically white men aged 40–44 years with incomes greater than $ 300.00. The third group was distinguished by the presence of periodontal pockets of 6 mm or more. The members of this group were typically adult females, black and mixed individuals who had 8 years or less of schooling, individuals with incomes ≤ $ 300.00 and widowers.

**Conclusion:**

The results suggest that periodontal health is worse in the group for which the social indicators are worse. Therefore, the social determinants of health also affect the severity of periodontal disease in adults Brazilian society.

## Background

Recently, increasing emphasis has been placed on the importance of economic, social and environmental factors in the understanding of oral diseases, and public health research has focused on the social determinants of health and disease. In particular, the interest in social determinants has increased with the recognition of the limitations of the traditional preventive approach in improving health and reducing social inequality [1].

In populations with low socioeconomic levels in developing countries, the prevalence of disease remains high compared with that in populations with higher socioeconomic levels [2] because the social conditions of a population are a determinant of health status [3]. This relationship must also apply to oral health , which is an integral and inseparable contributor to general health [4]. In addition, oral health is one of the health domains that can affect daily functioning and the general perception of health when pain and discomfort occur, which can cause several problems [5].

Pathological conditions are hypothesized to be associated with socioeconomic status. Those individuals who have a higher education level and greater purchasing power and live under more favorable conditions have better health statuses than those individuals who have lower education levels and live under less favorable conditions [6]. Thus, analyzing the relationships between social, economic and cultural factors and periodontal disease is of great importance because such analysis enables the development of public policies to improve the population’s health [7].

The fact that individuals with lower socioeconomic statuses have worse health indicators than those with higher socioeconomic statuses can also be applied to periodontal disease (PD): research has revealed an association between socioeconomic indicators and periodontal disease [8,9]. Although periodontal disease is more severe during adulthood, it should be emphasized that this disease is essentially progressive. It sometimes progresses slowly, and the age factor seems to be related to the duration of the disease rather than disease onset per se. Consequently, the higher incidence in adults simply reflects the longer period of time that local factors have contributed to the degradation of the tooth surface and to damage to the periodontal tissues (i.e., adults have had periodontal disease since their childhood or adolescence) [2].

Given that periodontal disease has a higher prevalence in adults [10,11] and that periodontal disease is one of the factors that cause edentulism [12,13] discover and describe its prevalence in the population and identify possible related factors may contribute to the reduction of dental mutilation.The present study aimed to describe the burden of periodontal disease among Brazilian adults and to identify possible relationships with the social determinants of health.

## Methods

A cross-sectional descriptive study using primary data from the southeastern region of Brazil was conducted from September to December 2010. The reference population for this study was comprised of adult males and females aged 35 to 44 years who lived in the urban area of a large city. Currently, nearly 96.9% of the population lives in urban areas [14], thus justifying the exclusion of rural areas (3.1%) from this study.

The Federal University of Minas Gerais Research Ethics Committee approved the present research project under protocol number 096/2009.

The selection of this age group was based on the recommendations of the World Health Organization (WHO); this age group is recommended for epidemiological surveys that assess the oral health of adults [15].

The sample size calculation was performed using the equation proposed by Lwanga and Lemeshow [16] to estimate disease prevalence. We adopted the following parameters: the prevalence of periodontal disease in the Brazilian adult population aged 35–44 years (34.6%) [11], a significance level of 5% (α = 0.05) and a margin of error of 20% (ϵ = 0.020). In this study ,in which the technique was to probabilistic sampling by clusters per stage correction was adopted for the design effect (deff) (2.0 was the highest possible) thus, the size of the final sample as multiplied by 2.0.The deff is a feature used to measure the effect of the sample plan on the average variance of the estimates by calculating the ratio of the estimated variance determined by the sampling plan to the estimate of the variance that would be obtained for a random sample of the same size [17].This procedure yielded a sample size of 832 subjects. The exclusion criteria were as follows: edentulism (21), refusal to participate (1), being bedridden (17), inability to answer the questions on the questionnaire due to a lack of understanding (20) and not being home during three contact attempts (30). Thus, the final sample consisted of 743 individuals. The total loss was 10.7%.

Adults of both sexes aged between 35 and 44 years who were present in the selected homes at the time of the interview and the exam and who agreed to participate in the study were included in the sample. The selection of the sample was based on the criteria adopted by the SB Brasil Project (Oral Health Conditions of the Brazilian Population) [10], which used a three-stage cluster sample with primary, secondary and tertiary sampling units. In operational terms, the sector was a territorial unit that was used to draw paths and identify households [18]. The primary units were census tracts made up of groups of homes. In general, each tract consisted of 300 homes, although the number varied according to the population density [19]. Based on the city map obtained from the *Instituto Brasileiro de Geografia e Estatística* (IBGE – Brazilian Institute of Geography and Statistics), 326 urban census tracts and 118 blocks could be identified in the city. A total of ten census tracts (primary sampling units) and 58 urban blocks (secondary sampling units) were randomly selected. Blocks and homes were randomly selected (1,450; third sampling stage), making replacements when necessary, until the number of adults needed for the sample was achieved.

The data collection was performed between May and December 2010. The data were obtained from interviews and epidemiological clinical exams conducted by trained dentists (inter-examiner reliability = 0.86 and intra-examiner reliability = 0.88). The examiners were properly dressed and used a mouth mirror, a WHO millimeter-scaled probe and a gauze sponges to perform the clinical exam. To pre-test the questionnaire, a test-retest procedure was performed using a group of 50 individuals over an interval of 15 days. In addition, a pilot study with 98 individuals was performed to assess the proposed methodology.

The following socioeconomic and demographic characteristics were analyzed: age (35–39 years and 40–44 years), sex (male, female), self-reported color (white, mixed, black, other), marital status (single, married, widowed, divorced/separated), per capita household income dichotomized by the median, so stay more homogenous groups, furthermore the group average was very close to the median (≤ $ 300.00 and > $ 300.00 per month) and education level (illiterate, 8 years, 9 to 12 years,> 12 years old).

The database construction and statistical analyses were performed using the Statistical Package for the Social Sciences (SPSS), version 19. A descriptive analysis of all study variables was performed. After this analysis, the researchers explored the relationships between periodontal disease and socioeconomic and demographic characteristics using multiple correspondence analysis. Multiple correspondence analysis is an exploratory technique used to analyze categorical data with a large number of variables and is used to visualize similar groups graphically.

## Results

In the present study, a total of 743 adults were examined and interviewed. The majority were female (69.2%), were between 35 and 39 years of age (52.0%), were of mixed color (49.7%), were married or cohabiting (71.7%),had less than eight years of education (55.6%) and had a per capita income lower than or equal to R$300.00 (51.7%) (Table 1).

**Table 1:** **Descriptive analysis of socioeconomic and demographic characteristics of Brazilian adults** (**N = 743)**

**Variables**	**N**	**%**
Age group		
35 a 39 anos	387	52,0
40 a 44 anos	357	48,0
Sex		
Male	229	30,8
Femele	514	69,2
Self-reported color		
White	187	25,1
Black	94	12,6
Mixed	369	49,7
Other	25	3,4
Did not respond	68	9,1
Marital status		
Married/Cohabiting	534	71,7
Separated/Divorced	60	8,1
Widowed	13	1,7
Single	129	17,5
Did not respond	7	0,9
Per capita household income		
≤ R$300,00	385	51,7
>R$300,00	322	44,4
Did not respond	36	4,8
Educação		
Illiterate	20	2,7
≤8 years	414	55,6
9 a12 years	251	33,7
> 12 years	53	7,9
Did not respond	5	0,6
CPI		
Healthy periodontium	279	37,5
Gingival bleeding	15	2,0
Calculus	360	48,4
Periodontal pockets of 4-5 mm	73	9,8
Periodontal pockets of 6 mm or more	16	2,1

The periodontal exams showed that 36.5% of the individuals had a healthy periodontium, 2.0% had gingival bleeding, 47.1% had calculus and 9.5% had periodontal pockets of 4–5 mm. The worst periodontal condition, which was periodontal pockets of 6 mm or more, was found in only 2.1% of the participants (Table 1).

**Figure 1:**
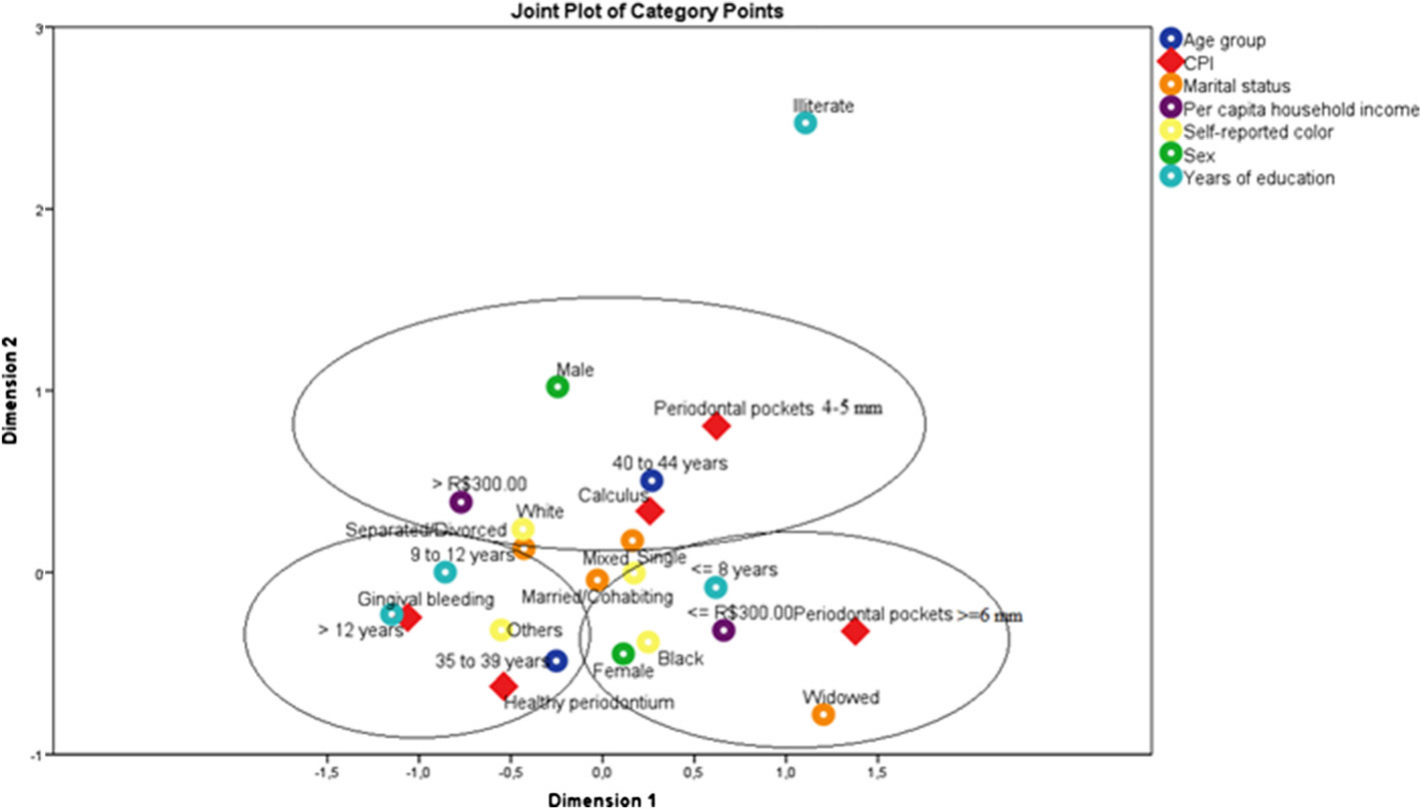
Profile of three groups formed by analysis multiple correspondence.

The correspondence analysis enabled us to form three groups with different profiles (Figure 1).

The relationships between the categories of the variables in this analysis were investigated without needing to assign a causal structure or assume a probability distribution a priori. This technique is appropriate for the study of population data, it is not inferential. This technique is useful when studying risk factors that may be associated with certain characteristics to be analyzed, and it allowed us to identify groups that have the same risk factors[20]. The first group (G1) was distinguished by the presence of bleeding (gingivitis) or a healthy periodontium. The members of this group were aged 35 to 39 years and had 9–12 years or more than 12 years of education. The members of the second (G2) had calculus or periodontal pockets of 4–5 mm. This group consisted of white men, aged 40–44 years with incomes greater than $ 300.00. The third group (G3) was distinguished by the presence of periodontal pockets of 6 mm or more. This group consisted of adult females, black and mixed individuals who had 8 years or fewer of schooling, individuals with incomes ≤ $ 300.00 and widowers.

## Discussion

The present study, performed in a large city in southeastern Brazil, aimed to explore the relationships between periodontal disease and socioeconomic and demographic characteristics (social determinants of health) using multiple correspondence analysis. In addition to collecting clinical data about periodontal conditions, we also conducted a survey focused on socioeconomic and demographic conditions at the individual level.

The majority of the participants in the study were females because the study was performed as a household study. Women continue to be home more often in Brazil and to be responsible for children. A higher proportion of females has been observed in epidemiological surveys that include exams performed in the home [21,22]. In addition, it should be emphasized that the life stage between the ages of 35 and 44 (the ages of those interviewed) is a productive period, and consequently, men and women are expected to be at work. However, in Brazil, many women stay home or perform work that enables them to remain at home for longer periods of time.

The majority of the adults in this sample had less than eight years of education although 3% of those studied were illiterate. The education level can reflect the social differences that coexist among individuals in the same situation with respect to vulnerability. In Brazil, the level of education of the population has increased. Between 2000 and 2010, the percentage of individuals without formal education or who had not completed primary school decreased from 65.1% to 50.2%; in addition, the percentage of individuals who had completed higher education increased from 4.4% to 7.9%. Education is a factor that affects the level of participation in political and social activities, and it can also improve or impair the health conditions of individuals [23].

The per capita household income was low. The results of the 2010 Demographic Census show that income inequality continues to be very high in Brazil, although there has been a decreasing trend in recent years. Although the mean per capita household income was R$668.00 in Brazil in 2010, 25% of the population received up to R$188.00, and half received up to R$375.00, less than one month of earnings at the minimum wage for that year (R$510.00) [23]. In the present study, more than half of the interviewees also earned less than one month of earnings at the minimum wage.

The periodontal condition was assessed using the Community Periodontal Index, which indicates the presence of bleeding (gingivitis), calculus and periodontal pockets. The researchers observed that the majority of the study population had a healthy periodontium. However, many had calculus (48.4%), and a small percentage of adults had gingival bleeding (2.0%) or periodontal pockets (11.9%). It should be emphasized that gingival bleeding is the first sign of periodontal disease, and it can be treated with simple measures. Moreover, gingival bleeding can be a marker of the health priorities of adults because it indicates the need for oral health guidance and prevention that can reduce gingival bleeding [24].

A small number of adults had shallow or deep periodontal pockets. This observation is in agreement with the findings of several national and international studies but differs from the findings of other studies, most likely due to the different methodologies used [11,25-28].

In a literature review of periodontal health conditions, based on the CPI, periodontal pockets ≥ 6 mm were found to affect from 10.0% to 15.0% of adults worldwide [29]. In another study performed in Germany with 925 adults aged between 35 and 45 years, the prevalence of periodontal pockets ≥ 4 mm among adults was 76.9%, with a higher prevalence among men [30].

In Brazil, the oral health condition survey, which used the CPI to assess the population according to macro-region and age group in 2010, showed that 1.9% of adults aged 35 to 44 had gingival bleeding, 28.6% had calculus, 15.2% had shallow pockets and 4.2% had deep pockets [11]. In the southeastern region, data from the SB Brasil Project (2010) revealed that 1.5% had bleeding, 30.5% had calculus, 16.7% had shallow pockets and 5.0% had deep pockets. In this survey, the prevalence of periodontal pockets was lower (11.9%).

Regarding the presence of calculus, it is clear that this condition is an indicator of poor oral hygiene and facilitates plaque buildup, which is an immediate cause of inflammation but not necessarily an indicator of the presence of disease [31]. However, calculus may contribute to a higher prevalence of bleeding because it represents a mechanical obstacle.

The joint analysis of the variables using multiple correspondence analysis indicated that the subjects could be divided into three groups. Differences that could cause health inequalities, which pose a challenge to be overcome in several countries, were observed among these groups [32]. Health inequalities can be defined as differences that cause certain social groups, such as the poorest individuals and ethnic minorities, to frequently face unequal conditions. This situation is reflected in the very poor health indicators of these groups [33].

The first group consisted of individuals with gingivitis and healthy periodontium. These adults had more years of education (9 to 12 years of schooling or more) and the youngest age (35–39 years).Gingival bleeding, which is reversible and easily controlled, was an indicator of disease found in this study. However, considering their level of education, these adults are more likely to be equipped to prevent diseases because they have better access to the information about oral disease prevention that is available in society [24,34]. These individuals are also more likely to attend preventive or follow-up visits because socioeconomic characteristics (such as income and level of education) influence the pattern and type of dental services used [35].

Group two (G2) was characterized by the presence of shallow pockets (4–5 mm) and calculus. This group consisted of white men with a higher per capita income and an age range of 40 to 44 years. Interestingly, this group had a better social status, with incomes that allowed both the purchase of oral care products and a better diet. Therefore, these participants had a greater chance of developing good oral health habits. This group was characterized by the presence of men. We know that gender is a factor that can play an important role in the health/disease due to health behaviors [36-39]. Another factor to be considered in this group is the oral hygiene standards because the build-up of calculus is indicative of a small number of visits to the dentist. Moreover, not only sex but also age may influence periodontal health. It has been observed that individuals between 40 and 50 years old have worse periodontal health [40].

Group three (G3) was characterized by the presence of deep pockets (6 mm or more) and advanced periodontitis. This group calls attention to factors that may have negative effects on health: black or mixed color with a lower income (≤ 300.00), low education level (≤ 8 years) and being widowed. The composition of this group emphasizes the socioeconomic, demographic and cultural aspects of a marginalized society, demonstrating that systematic inequalities in health positions groups of people who are already socially underprivileged to be disadvantaged even more with respect to health, i.e., they further increase the social differences that generate inequity [41]. The women were also in this group.

This group provides evidence that the social determinants of health are related to periodontal disease [42]. In addition to educational level and socioeconomic status, race was also associated with periodontitis. At least one study has found that black individuals, especially individuals with low educational levels and those living in neighborhoods with poor socioeconomic statuses, were more likely to have periodontal disease [8]. Black individuals and individuals of mixed color likely have a greater chance of developing periodontal disease due to their worse economic situations, which hinder access to dental treatment and information, favoring the development of oral health problems .Biological susceptibility to periodontitis between different races was not especially evident, although an American study found that black Americans had more chance of developing periodontal disease than white Americans [25]. It is possible that skin color is associated with periodontal disease independent of social class and biological characteristics, with black and mixed race individuals being exposed to greater stress, which is a risk factor for periodontal disease [31]. The study by Borrell et al. [43], however, demonstrated that there are relationships between periodontal disease and both socioeconomic differences and race/ethnicity. After adjustment for confounding factors, the combined effect of higher education and higher income resulted in significantly better outcomes in relation to periodontitis. Education and income were independently associated with periodontitis, and there was a significant inverse relationship for each racial/ethnic group. The combined effect of higher education and higher income resulted in higher levels of periodontitis among non-Hispanic whites and Mexican Americans but not among non-Hispanic blacks. Non-Hispanic blacks with high education levels and high incomes had a prevalence of periodontitis that was similar to that of non-Hispanic blacks with low education levels and low incomes. However, it is important to note that some studies have shown that the relationship between socioeconomic indicators and poor neighborhood have little or no influence on health [44-46].

It should be emphasized that the most severe form of periodontal disease (pockets ≥ 6 mm), which requires treatment and specialized dental follow-up to ensure that these pockets do not cause tooth loss, was found in Group 3 [47,48]. Although the women in this group had deep periodontal pockets, previously published studies indicate that women have better oral hygiene than men and visit the dentist more often. This factor is more important than any genetic factor [6]. In addition, women perceive health problems more accurately [49]. Periodontal disease, although found in more women in this study, may be linked more closely to socioeconomic factors than to gender.

It is necessary to emphasize that the correspondence analysis did not match the number of individuals in the sample but did match the distribution of the categories of each variable with respect to the dependent variable [20], which was periodontal disease. The relationships between the categories of variables investigated in the groups targeted for this study had no a priori claim to assume a probability distribution but were used identify the profiles of groups that have the same risk factors that may be associated with certain characteristics, such as the factors analyzed in this study.

The present study measured periodontal conditions using the CPI. This instrument is limited because it provides only a partial measure of the disease and does not take its historical evolution into consideration. The CPI can also underestimate the prevalence of this disease, it is less severe because bleeding is not considered after calculus or periodontal pockets are identified [50] (i.e., the CPI does not identify the actual prevalence of this less severe condition because the activity of the disease is not considered in these cases) [51].

Another limitation of the instrument used to diagnose periodontal disease is the difficulty in defining the condition of the periodontium because of its complexity. An individual can have different areas with distinct levels of severity, varying from healthy to deep periodontal pockets [23]. To reduce this problem, all of the teeth were examined in this study.

In a previous study [52] that used the CPI to assess the prevalence of periodontal disease in a group of individuals whose data were obtained from a full-mouth exam using partial records that depicted half of the mouth and index teeth, it was observed that half-mouth exams can be used in cross-sectional studies without distorting the prevalence of periodontal disease. However, the data obtained from index teeth cannot be used to assess the extent or prevalence of periodontal disease because these data exhibit deviations that can affect the results.

## Conclusions

In the present study, the socioeconomic and demographic characteristics were related with periodontal disease. The female adults in G3 had characteristics of iniquity, such as low incomes, fewer years of education, black or mixed color and advanced periodontitis.

## Competing interests

The authors declare there are no conflicts of interest related to the present study.

## Authors’ contributions

MLCB was responsible for the acquisition of the data, the analysis and interpretation of the data and the organization and drafting of the paper. ACVC was responsible for data analysis and interpretation. AMDV, FFM and EFF were responsible for the study supervision during data collection and assisted with the data analysis and interpretation, thus contributing critically to the progress of the study. All authors approved the final version to be published, in addition to reading and approving the final manuscript.

## Pre-publication history

The pre-publication history for this paper can be accessed here:

http://www.biomedcentral.com/1472-6831/13/22/prepub
